# Pasteurella multocida Bacteremia in an Immunocompromised Patient With Esophageal Squamous Cell Carcinoma: A Case Report

**DOI:** 10.7759/cureus.75924

**Published:** 2024-12-18

**Authors:** Rafaela Lopes Freitas, Fábio Reis, Eduarda Ruiz Pena

**Affiliations:** 1 Internal Medicine Service, Pedro Hispano Hospital - Matosinhos Local Health Unit, Matosinhos, Porto, PRT; 2 Infectious Diseases Service, Pedro Hispano Hospital - Matosinhos Local Health Unit, Matosinhos, Porto, PRT

**Keywords:** bacteremia, immunocompromised host, pasteurella multocida, pneumonia, zoonotic infections

## Abstract

*Pasteurella multocida (P. multocida)* is a facultative anaerobic Gram-negative coccobacillus that represents a rare cause of systemic infection in immunocompromised patients.

This report presents the case of a 59-year-old man with advanced squamous cell carcinoma of the oesophagus, recently undergoing radiotherapy and chemotherapy, halted due to cytopenias, including neutropenia. The patient, who owned a cat but denied any recent bites or scratches, developed bacteremia caused by *P. multocida* with presumed pulmonary and renal foci. Early diagnosis and prompt treatment with intravenous cephalosporins led to clinical improvement.

This case highlights the need to consider zoonotic pathogens in vulnerable patients and the importance of early therapeutic intervention. A brief review of current literature on *P. multocida* is included, underscoring diagnostic challenges and treatment approaches for this rare entity.

## Introduction

*Pasteurella multocida* (*P. multoida*) is an opportunistic pathogen commonly isolated from the oral and respiratory tracts of cats and dogs [1,2]. While animal bites and scratches are the primary transmission routes, indirect exposure, including close contact with pets, has been implicated in rare systemic infections.

The organism predominantly causes localised infections, such as cellulitis, but can occasionally progress to systemic involvement, particularly in immunosuppressed individuals [3]. In patients undergoing treatment for malignancies, factors like neutropenia, mucosal barrier disruption, and generalised immune dysfunction increase susceptibility to invasive infections [4-7]

This case report highlights a rare presentation of *P. multocida* bacteremia in a patient with advanced oesophageal squamous cell carcinoma. Pulmonary and renal involvement were presumed based on clinical and imaging findings. The case is discussed in the context of current literature, with a focus on diagnostic and therapeutic considerations.

## Case presentation

A 59-year-old man, a former smoker (66 pack-years), was diagnosed with squamous cell carcinoma of the cervical and thoracic oesophagus (cT4N1M0) in the present year. He was undergoing radiotherapy and chemotherapy; however, treatment was interrupted after four cycles due to the development of grade 2 thrombocytopenia and grade 3 neutropenia consistent with treatment-related toxicity. He had no significant surgical history or family history. The patient owned a cat but denied any recent bites or scratches.

The patient presented to the emergency department with a three-day history of fever (38.7°C), fatigue, dyspnoea, pleuritic chest pain, and productive cough. On physical examination, his vital signs included fever (39.3°C), hypotension (blood pressure of 95/60 mmHg), and tachycardia (heart rate of 115 bpm). A respiratory exam revealed poor air movement with diminished breath sounds at the bases bilaterally and no audible wheezing or crackles. There were no signs of focal skin lesions or bite wounds.

Arterial blood gas on room air showed pH of 7.51, partial pressure of carbon dioxide (pCO2) of 33 mmHg, and partial pressure of oxygen (pO2) of 60 mmHg. The chemistry panel was significant for lactic acid 3.0 mmol/L. All other values were within normal limits.

Laboratory findings on admission revealed neutrophilia (7 x 10^3/uL) and elevated inflammatory markers (C-reactive protein of 423 mg/L, ferritin 769.89 ng/mL) (Table 1).

**Table 1:** The patient's laboratory findings on admission

Test	Result	Reference range
Haemoglobin	12.1 g/dL	13 – 18 g/dL
White blood cell count	8.09 x 10^3^/uL	4 – 11 x 10^3^/uL
Neutrophil count	7.0 x 10^3^/uL	1.3 – 6.8 x 10^3^/uL
Platelets	200 x 10^3^/uL	150 – 400 x 10^3^/uL
C-reactive protein	423 mg/L	<5.0 mg/L
Pro-calcitonin	0.79 ng/Ml	< 0.5 ng/mL
Ferritin	769.89 ng/mL	15-200 ng/mL
Creatinine	0.7 mg/dL	0.7 - 1.3 mg/dL
Urea	23 mg/dL	18 - 55 mg/dL
Sodium	137 mEq/L	135 – 145 mEq/L
Potassium	3.9 mEq/L	3.4 – 5.1 mEq/L
Alanine aminotransferase (ALT)	8 U/L	< 55 U/L
Aspartate aminotransferase (AST)	23 U/L	5.0 - 34 U/L
Gamma-glutamyl transferase (GGT)	22 U/L	< 55 U/L
Alkaline phosphatase (ALP)	79 U/L	40 – 150 U/L

A chest radiograph demonstrated bilateral patchy opacities suggestive of an infectious process. No pleural effusion or pneumothorax was identified. Blood and urine cultures were obtained along with a respiratory virus and influenza panel and urine streptococcal and legionella antigens. The patient was initially treated with intravenous (IV) ceftriaxone and azithromycin for empirical antibiotic coverage, as community-acquired pneumonia was assumed, and was subsequently admitted.

Blood cultures grew *P. multocida* within 48 hours, which was sensitive to all tested antibiotics, including amoxicillin-clavulanate, doxycycline, cefotaxime, and penicillin. Additionally, a strain of *Klebsiella pneumoniae* was isolated from bronchial secretions, exhibiting susceptibility exclusively to ceftriaxone. All other cultures and viral panels returned negative. Therefore, the patient’s treatment was de-escalated to ceftriaxone monotherapy.

**Figure 1:**
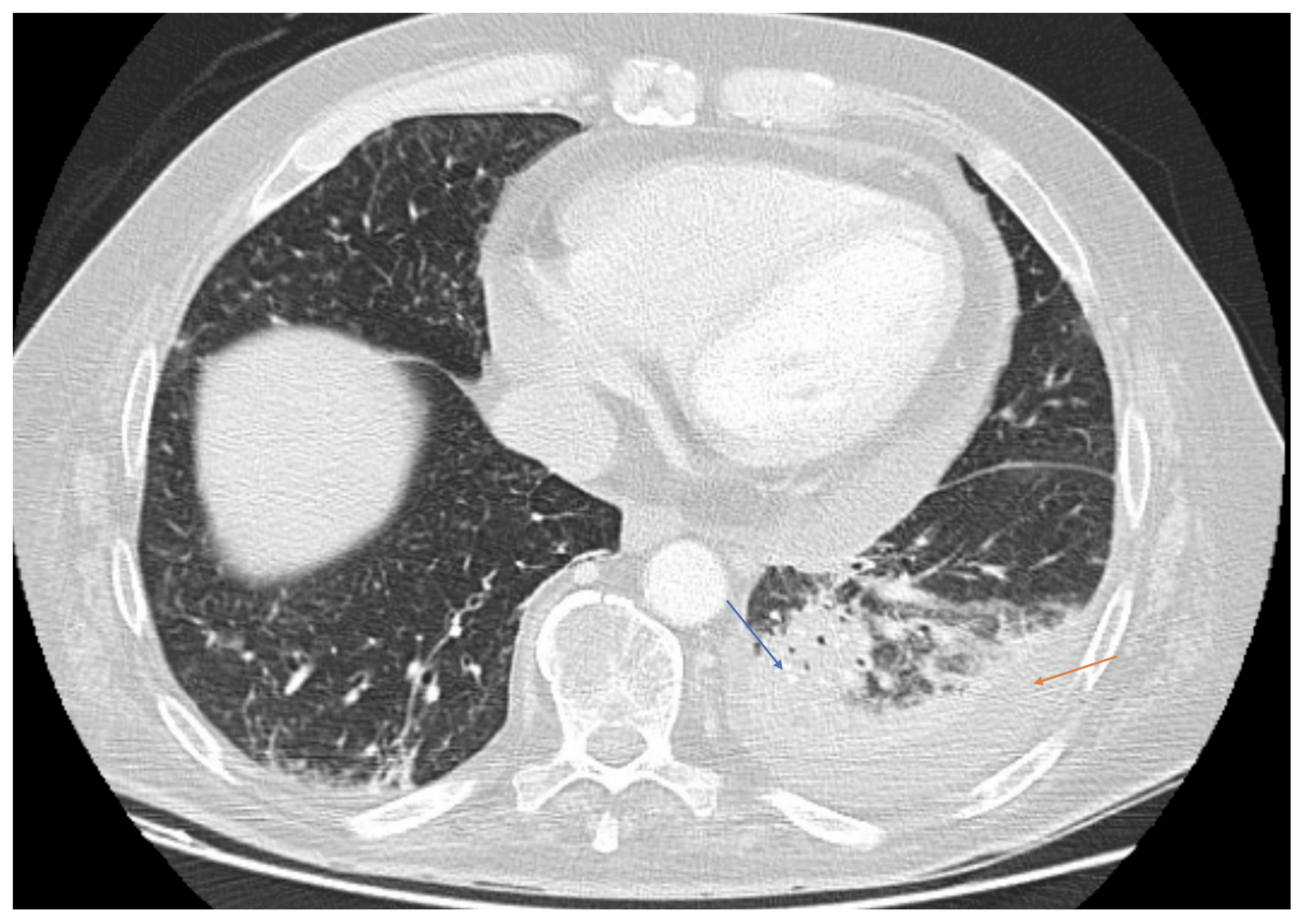
Contrast-enhanced CT scan of the chest showing alveolar infiltrates in the lower lobe of the left lung (blue arrow) with adjacent pleural effusion (orange arrow)

**Figure 2:**
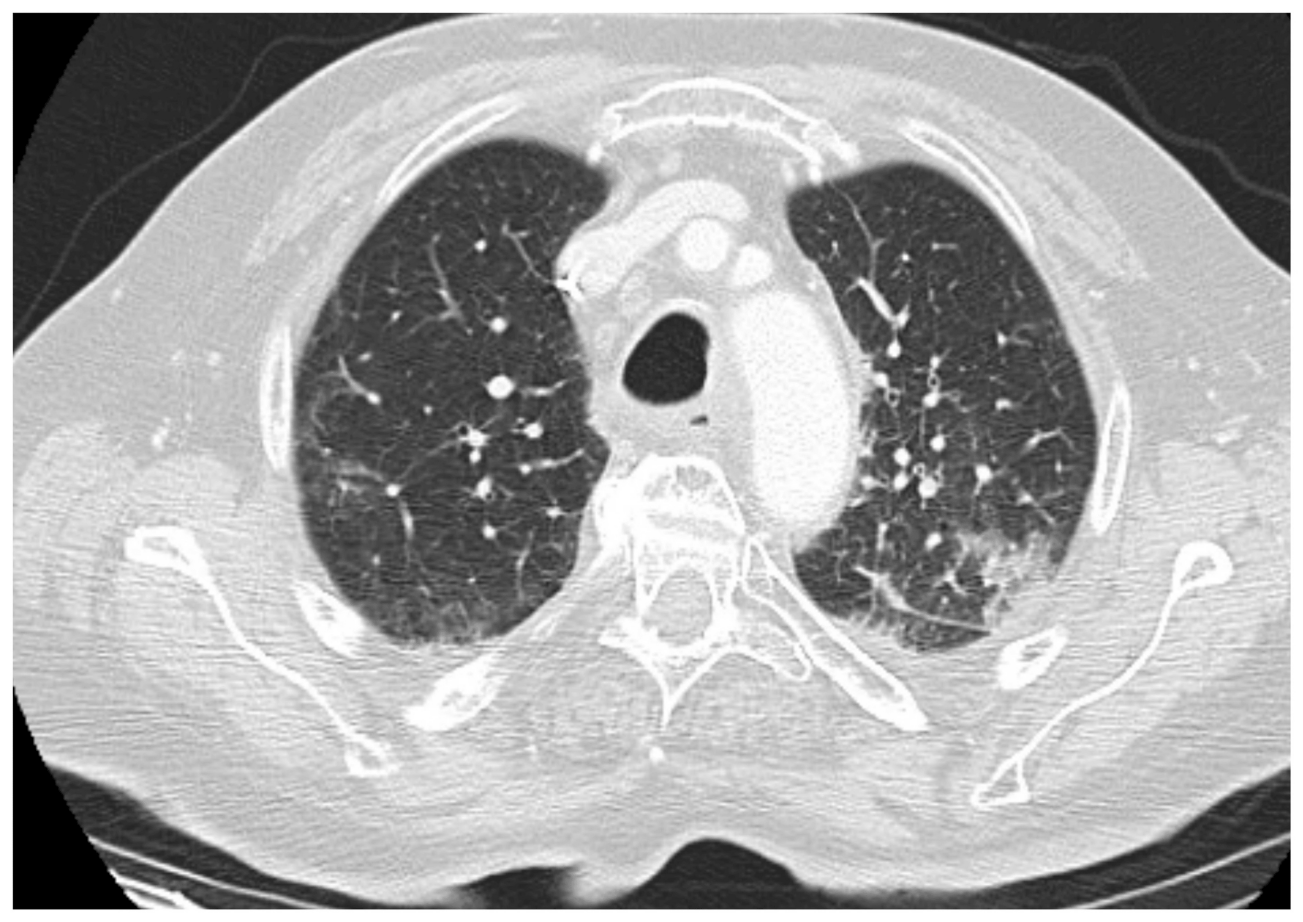
Contrast-enhanced CT scan of the chest demonstrating multiple tree-in-bud opacities and peribronchial consolidations

**Figure 3:**
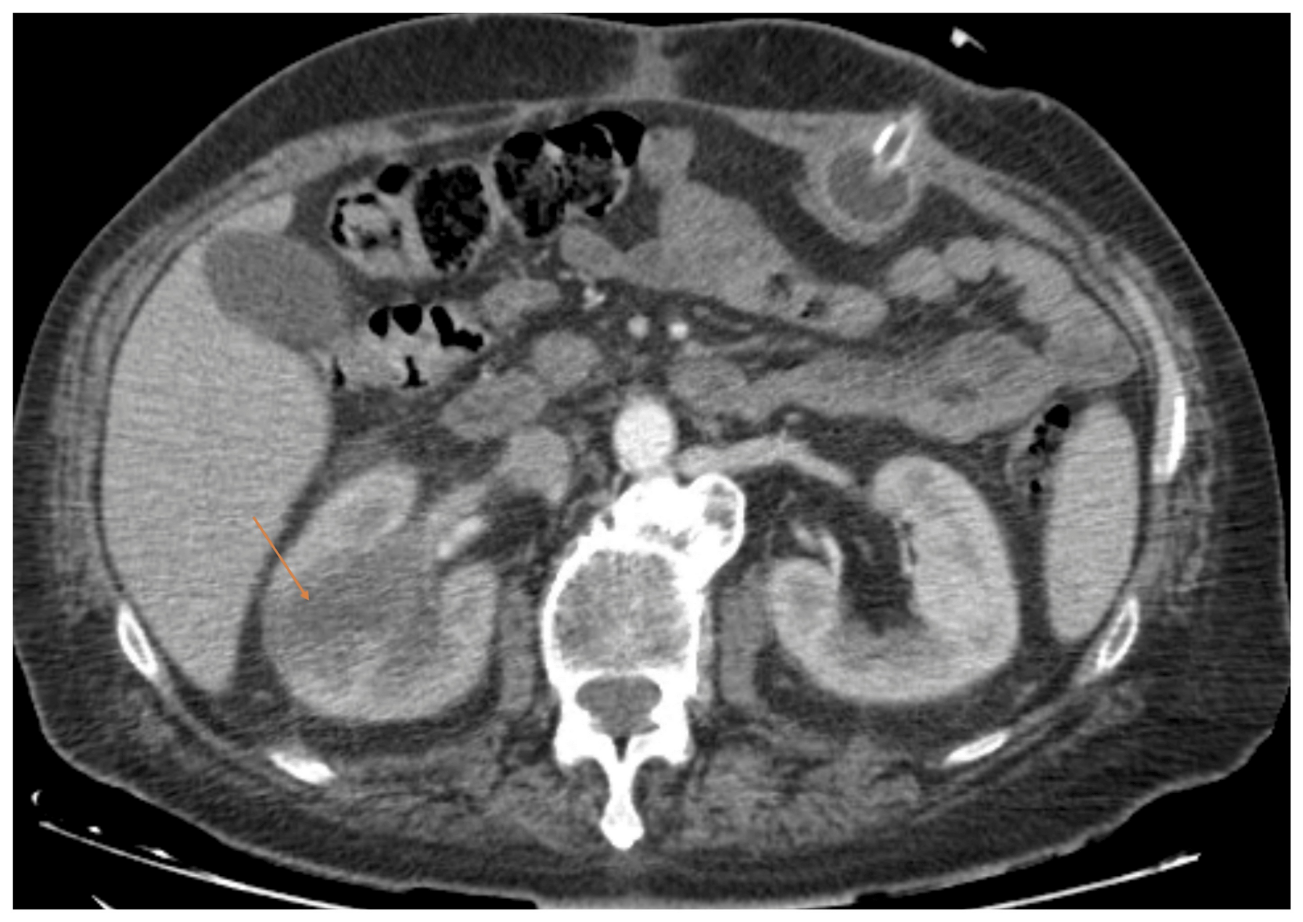
Contrast-enhanced CT of the abdomen showing the right kidney with hypodense lesions (orange arrow) consistent with multifocal pyelonephritis

The contrast-enhanced computed tomography (CT) findings were significant for multifocal pneumonia within an alveolar infiltrate in the lower lobe of the left lung, with a small adjacent pleural effusion (Figure 1). In the posterior segment of the left upper lobe, the posterior segment of the right upper lobe, the middle lobe, and the right lower lobe, multiple tree-in-bud opacities and peribronchial consolidations were observed, likely of infectious origin (Figure 2). The right kidney showed hypodense lesions (the largest measuring 5 cm), suggestive of multifocal pyelonephritis (Figure 3). There was no evidence of neoplastic progression.

The patient was diagnosed with disseminated *P. multocida* infection, presenting with bacteremia and probable renal and pulmonary involvement.

Clinical improvement was observed within 72 hours following the initiation of IV antibiotic therapy, with resolution of fever, stabilisation of vital signs, and normalisation of inflammatory markers. New blood cultures after 48 h of IV treatment were negative.

The patient was successfully discharged after completing 14 days of intravenous ceftriaxone, with a recommendation to continue oral cefixime for an additional 14 days to complete the treatment course.

## Discussion

*Pasteurella multocida* is a Gram-negative, facultative anaerobic coccobacillus commonly found as part of the normal flora in the upper respiratory tract of domestic animals, particularly cats and dogs [8]. It is also recognised as an important zoonotic pathogen. Studies estimate that up to 90% of cats and 50% of dogs carry this microorganism, with transmission to humans occurring through bites, scratches, or close contact. While most human infections are localised, systemic manifestations such as bacteremia are rare and typically occur in immunocompromised individuals [9].

The case described highlights the role of neutropenia and disrupted mucosal barriers in facilitating systemic infection. The patient’s close contact with animals was a key risk factor, reflecting findings from the literature, which underscore the link between zoonotic exposure and *P. multocida* infections. Immunocompromised individuals, particularly those with malignancies, diabetes, chronic liver disease, or undergoing chemotherapy, are at heightened risk due to impaired neutrophil function and reduced mucosal barrier integrity [9,10]. The organism’s virulence is mediated by mechanisms such as its polysaccharide capsule, which resists phagocytosis, and lipopolysaccharides that trigger inflammatory cascades. Additionally, the production of proteases that degrade host immunoglobulins further compromises immune defences.

The clinical spectrum of *P. multocida* infections is broad, ranging from soft tissue infections, often following animal bites, to respiratory tract infections, septic arthritis, osteomyelitis, and bacteremia. Although bacteremia is rare, it carries significant morbidity and mortality in immunocompromised patients [11, 12]. Pulmonary and renal involvement, as suspected in this case, are particularly uncommon. Pulmonary infection may arise from aspiration, haematogenous spread, or contiguous extension, while renal involvement may occur through haematogenous dissemination [13].

Diagnosis of *P. multocida* bacteremia can be challenging due to its non-specific presentation and the rarity of systemic involvement. Blood culture remains the diagnostic gold standard, with growth typically observed within 24 to 48 hours. However, the zoonotic origin of *P. multocida *infections is frequently overlooked unless a detailed history of animal exposure is obtained. In this case, the patient’s close contact with a household cat provided an essential diagnostic clue. Imaging modalities, such as CT, are valuable in identifying focal organ involvement. Findings such as pulmonary opacities and pyelonephritis in this case emphasise the importance of a multimodal diagnostic approach.

Treatment of *P. multocida *infections relies on prompt initiation of effective antibiotic therapy. The organism is generally sensitive to beta-lactams, which remain the first-line agents [10]. Resistance, although rare, has been documented for macrolides and aminoglycosides. In this case, the patient responded well to intravenous ceftriaxone, with clinical improvement noted within 48 hours. Transitioning to oral amoxicillin-clavulanate facilitated complete recovery while reducing hospital stay. Current guidelines recommend treatment durations of 10 to 14 days for uncomplicated bacteremia, with longer courses indicated for focal complications such as endocarditis or osteomyelitis. In this case, the authors have chosen a 28-day course of antibiotic therapy to ensure adequate penetration into the infected tissues and to address potential complications, such as abscess formation.

Regarding prognosis, systemic *P. multocida* infections in immunosuppressed patients are associated with significant mortality, particularly when diagnosis is delayed or treatment is inappropriate. Mortality rates approach 30% in bacteremia cases, with malignancy being a significant risk factor. Timely recognition and early initiation of targeted therapy are critical to improving outcomes. In this case, the favourable outcome underscores the importance of clinical suspicion for zoonotic infections in immunocompromised populations.

The development of literature on *P. multocida* highlights the need for further research into its pathophysiology, particularly in immunocompromised individuals. Recent studies suggest that the organism’s ability to evade immune defences involves multiple factors, including capsule-mediated immune modulation and biofilm formation on host tissues. Gaps remain in understanding optimal antibiotic regimens and the role of prophylactic measures for high-risk patients. Future research should prioritise the development of clinical algorithms for early diagnosis, risk stratification, and tailored management strategies for *P. multocida* infections.

## Conclusions

This report highlights a rare case of *P. multocida *bacteremia in an immunocompromised patient with advanced oesophageal carcinoma. Prompt recognition of zoonotic pathogens and initiation of targeted therapy were pivotal to the favourable outcome. Clinicians should consider *P. multocida* in febrile immunocompromised patients, particularly those with animal contact, and initiate appropriate antimicrobial therapy early to prevent complications.
